# A debug scheme to improve the error identification in post-silicon validation

**DOI:** 10.1371/journal.pone.0202216

**Published:** 2018-09-04

**Authors:** Inhyuk Choi, Won Jung, Hyunggoy Oh, Sungho Kang

**Affiliations:** Department of Electrical and Electronic Engineering, Yonsei University, Seoul, Korea; University of Waterloo, CANADA

## Abstract

While developing semiconductors, post-silicon validation is an important step to identify the errors that are not detected during the pre-silicon verification and manufacturing testing phases. When the design complexity increases, the required debug time also increases because additional debug data are required to identify the errors. In this study, we present a debug scheme that improves the error identification capability. The proposed debug approach concurrently generates three types of signatures using hierarchical multiple-input signature registers (MISRs). The error-suspect debug cycles are determined by analyzing the debug cycles that are commonly contained in the erroneous signatures of the three MISRs. To reduce the amount of debug data, we compare the high-level MISR signatures in real time with the golden signatures; further, we handle the remaining two MISRs based on the tag bits that are obtained from the results of the high-level MISR. The experimental results prove that the proposed debug structure can significantly improve the error identification capability using less debug data than that used in previous debug structure.

## Introduction

Recent advances in semiconductor manufacturing technologies have caused the development of improved designs that contain faster and more diverse functions. However, these developments have also enabled the creation of more complex designs that require additional logic. To produce these complex designs, an additional effort is required on the part of the designers [1]. The incorporation of simulation models into the design process is becoming increasingly insufficient to perform error detection because both the size and the complexity of the integrated circuit designs have increased [2]. Errors that were not detected during the pre-silicon verification stage or the manufacturing testing stage will appear in the first silicon. To eliminate these errors, designers attempt to rapidly identify these errors and apply solutions, such as software changes, engineering change orders, and the usage of spare cells [3]. If design modifications are required to solve various problems, this process should be completed before re-spinning the silicon. However, silicon re-spinning causes an increase in the production costs and impacts the time-to-market [2]. Therefore, post-silicon validation has been recognized as an important step to manufacture semiconductors, and various related debug methods have been studied and introduced.

Based on the controllability of error occurrence, post-silicon debug is generally classified into two phases: nondeterministic and deterministic [4, 5, 6]. In the nondeterministic phase, the main objective is to control intermittent, transient, and electrical errors to facilitate the design-for-debug (DFD). If the errors are controllable, they are identified to be deterministic and repeatable events. Using DFD techniques, it is possible to detect the root cause of controllable errors. One important concern while using the DFD technique for post-silicon validation is related to the determination of the number of debug cycles that can be observed in a circuit under debug (CUD). If there is an increase in the number of observed debug cycles, it can cause clearer error identification. Debug techniques using trace buffers are currently being employed to achieve high observability that is in accordance with the observation of consecutive debug cycles. However, the number of consecutive debug cycles is limited by the size of the trace buffer [7]. Therefore, some debug techniques have applied a multiple-input signature register (MISR) to compact the debug cycles and to observe the generated signatures [8, 9, 10, 11]. The signatures generated by MISR are unloaded to an external debugger and compared with the golden signatures to determine whether the signatures are erroneous. In this case, the golden signatures are generated using the verified data in the pre-silicon step. An example of the external debug interface is the test access port (TAP) of IEEE 1149.1 between the CUD and the external debugger. Regardless, the exact erroneous debug cycles cannot be detected by observing the signatures. Therefore, repeatable debug sessions that decrease the compaction ratio of an MISR signature are required until the observed debug cycles are obtained in a raw format. In this case, the number of debug data increases because of the repeatable debug sessions; further, the debug time also increases even though we have ensured efficient error identification. To address the problems that are related to the number of debug data and repeatable debug sessions, the debug techniques in [12, 13] use an MISR and a cycling register to compact the debug cycles using different compaction policies; the MISR compacts the consecutive debug cycles, and the cycling register compacts the debug cycles at regular intervals. When this is performed, the debug cycles that are commonly contained between erroneous MISR signatures and the cycling register are analyzed as error-suspect debug cycles. Further, the exact erroneous debug cycles are identified by observing the error-suspect debug cycles of CUD again. Although the number of debug sessions and debug data are dramatically reduced using this debug technique, the non-erroneous debug cycles can be analyzed as error-suspect debug cycles. Further, the debug time still increases because of the existence of unnecessary debug data during the identification of the exact erroneous debug cycles.

In this study, we propose an improved debug scheme to reduce the amount of debug data. Our proposed debug scheme is based on hierarchical compaction using three MISRs. The usage of multiple hierarchical MISRs increases the observability and reduces the number of error-suspect debug cycles in case of error identification. Additionally, a reduction in the amount of debug data causes a reduction in the debug time.

## Previous work and motivation

The debug scheme in [11] was proposed to reduce the debug time by comparing with [9]. In the debug work in [11], the main debug framework is based on the repeatable debug sessions introduced in [9]. In the debug approach in [11], the MISR compaction ratio is reduced as the debug session is progressed in a similar manner as [9]. In this case, the golden signatures are pre-stored in the trace buffer before the debug session. By applying the pre-stored golden signatures, the on-chip comparison between the golden signatures and the reference signatures generated by the MISR is facilitated. The area which the pre-stored golden signatures are located is empty after the on-chip comparison. At the same time, the additional MISR compacts the trace cycles by the segmented compaction ratio. If the result of the on-chip comparison denotes that the generated MISR signature is error-suspected, the segmented MISR signatures by the additional MISR compaction are stored in the empty area of the trace buffer. Therefore, the consecutive debug sessions are performed simultaneously, then the overall debug time can be reduced by comparing with [9]. Nevertheless, the debug approaches in [9, 11] are unsuitable for nondeterministic debug phase because a number of repeatable debug sessions are required to observe the error-suspect interval. For this reason, these debug approaches focus on only the deterministic debug phase and the cycle-accurate error occurrence.

To reduce the amount of debug data, the debug method proposed in [12] reduces the repeatable debug sessions and analyzes the error-suspect debug cycles using the MISR and the cycling register. In this technique, the signatures generated by the MISR and the cycling register are captured in the trace buffer and are unloaded to the external debugger; further, the generated signatures are compared with the golden signatures. The signatures generated by the MISR are used to compact the consecutive debug cycles, whereas the signatures generated by the cycling register are used to compact the debug cycles at regular intervals. The erroneous signatures of the MISR and the cycling register are sorted by comparing with the golden signatures. It is not possible to detect the exact erroneous debug cycles by observing the erroneous signatures; therefore, the error-suspect debug cycles that simultaneously affect the corruption of the signatures generated by the MISR and the cycling register has to be analyzed. In this case, the error-suspect debug cycles are determined to be the commonly contained debug cycles in both the MISR signatures and the cycling register. After analyzing the error-suspect debug cycles, the debug session is repeated to capture the error-suspect debug cycles in a raw format into the trace buffer. To selectively capture the error-suspect debug cycles, tag bits that provide the positions of the error-suspect debug cycles are generated with lossy compression and are uploaded to the trace buffer. The error-suspect debug cycles are captured based on the tag bits. To analyze the exact number of erroneous debug cycles, the captured error-suspect debug cycles are unloaded to the external debugger and are compared with the golden debug cycles. The debug technique proposed in [12] is insufficient to efficiently filter out the error-free debug cycles in the error-suspect debug cycles as the length of the observation window is widen. Therefore, the debug data, such as the error-suspect debug cycles and tag bits, are increased to identify the exact erroneous debug cycles. Increment in the debug data causes increment in debug time because the debug data are transferred via external debug interfaces that are operated at low operational frequencies. Additionally, a number of tag bits are required to capture the error-suspect debug cycles in a raw format because of the usage of lossy compression. Therefore, many debug cycles should be analyzed in order to pinpoint the exact erroneous cycles, and this increases the debug time.

The debug technique in [13] is proposed to reduce the quantity of debug data by comparing with [12]. In the debug work in [13], the tag map is applied to reduce the number of tag bits without lossy compression. Nevertheless, the MISR and the cycling register compact the debug cycles similar with the debug technique in [12]. In this case, reduction of the number of required debug data to identify the erroneous cycles is limited. Additionally, the length of observation window is also restricted though the number of tag bits is reduced. Therefore, the effect of reduction in debug time is insignificant by applying the debug approach in [13].

**Fig 1:**
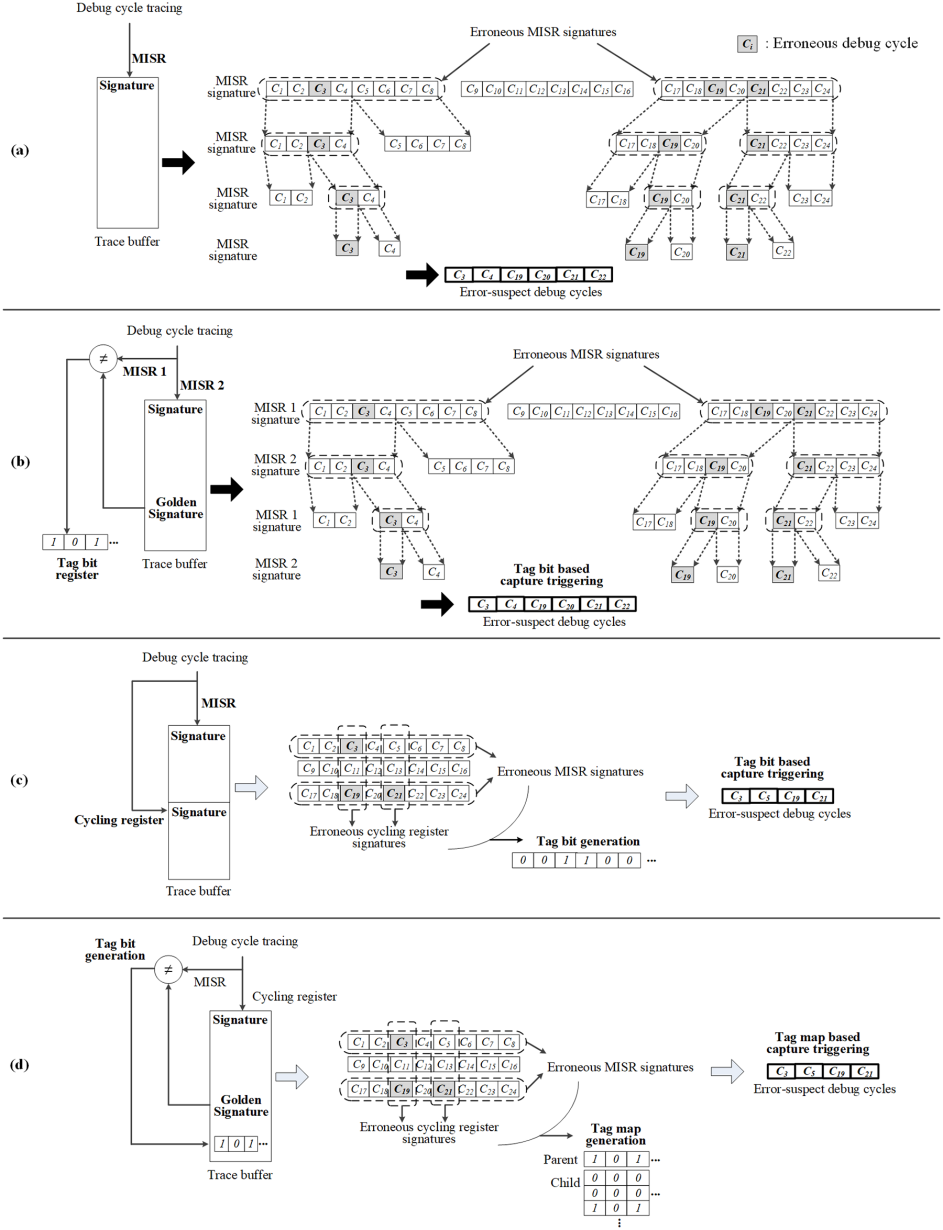
The previous debug techniques: (a) debug technique in [9]; (b) debug technique in [11]; (c) debug technique in [12]; (d) debug technique in [13].

Fig 1 summarizes the previous debug techniques in [9, 11, 12, 13] in accordance with the usage of the signatures and the identification of the error-suspect debug cycles.

We propose a debug scheme that applies hierarchical compaction using multiple MISRs and that improves the error identification capability in comparison with that used in the previous techniques. In order to tackle the results of the previous works, the proposed debug scheme supports not only the deterministic debug phase but also the nondeterministic debug phase. Because the error occurrence can be varied during multiple debug runs in the nondeterministic environment, reducing the debug runs is efficient to adapt the nondeterministic environment. In the proposed debug scheme, the error-suspect debug cycles are identified after the first debug run. On the contrary, repeatable debug runs are required to observe the error-suspect interval for the identification of the error-suspect debug cycles in the debug techniques in [9, 11]. In this case, the hit rate in the variation of the error occurrence is improved in the proposed debug scheme compared with [9, 11]. At the same time, the number of required debug data to identify the erroneous cycles is also reduced to contribute for the reduction of debug time compared with [12, 13].

In the proposed debug scheme, the high-level MISR signatures are compared with the golden signatures in the trace buffer using an on-chip comparator to predecide the error-suspect debug intervals. The remaining two low-level MISRs are used to selectively compact the debug cycles based on the result of predecision in the high-level MISR. The commonly contained debug cycles among the low-level MISR signatures are determined as the error-suspect debug cycles. In this case, the number of commonly contained debug cycles can be reduced because the length of the error-suspect intervals to generate the low-level MISR signatures is reduced by the on-chip comparison using high-level MISR structure. Thus, we can effectively reduce the number of unnecessary error-suspect debug cycles that are required to identify the erroneous debug cycles. At the same time, the required number of debug sessions to observe the error-suspect intervals is reduced in the overall debug operation. For this reason, the proposed debug scheme can be adapted to the variation of the nondeterministic error occurrence during the debug operation. Additionally, we apply a tag map to reduce the number of tag bits in the proposed debug scheme. Therefore, the debug time also decreases because the amount of debug data required for error identification is less than that required in case of the previous debug techniques.

## Proposed debug scheme

Starting with the overall debug process, this section presents the proposed debug scheme in detail. The proposed debug scheme contains the debug operation and the debug structure.

### Concept of the proposed debug process

**Fig 2:**
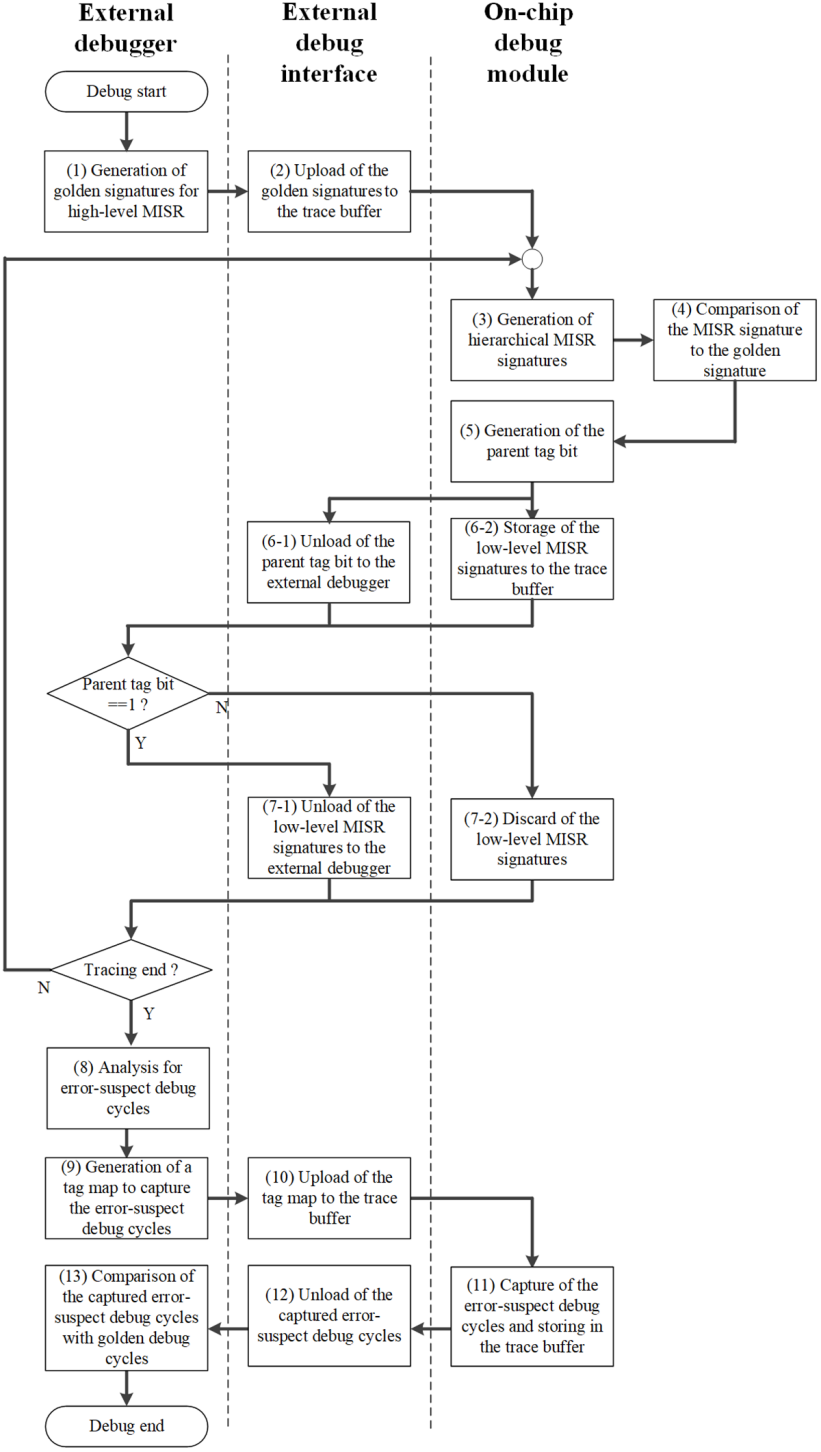
Operational flow of the proposed debug scheme.

Fig 2 depicts the operational flow of our proposed debug scheme. The scheme includes an external debugger, an external debug interface, and on-chip debug module operations. In the external debugger, the golden signatures for the high-level MISR are generated by presimulating CUD (1). In this state, we determine the size of the observation window, i.e., the number of debug cycles to be observed. Further, we upload the golden signatures to the on-chip trace buffer via the external debug interface (2). The uploaded golden signatures are compared with the high-level MISR signatures that are generated during the real-time tracing of the debug cycles. Low-level MISR signatures are also generated simultaneously (3). By comparing the high-level MISR signatures and the golden signatures (4), the parent tag bit is generated (5). The parent tag bit is unloaded to the external debugger to notify the existence of the erroneous high-level MISR signature (6–1). At the same time, the low-level MISR signatures are stored in the trace-buffer (6–2). Further, the stored signatures are unloaded to the external debugger by referring to the values of the parent tag bit (7). After the observation of the overall debug cycles is completed, the error-suspect debug cycles are analyzed in the external debugger (8). After completing the analysis, we generate a tag map to selectively capture the error-suspect debug cycles (9). Further, the tag map is uploaded to the trace buffer (10), and the error-suspect debug cycles are selectively captured in the trace buffer based on the tag map (11). In this case, the error-suspect debug cycles can be observed until the trace buffer is full. After capturing the error-suspect debug cycles, the captured debug cycles are unloaded via the external debug interface (12). The unloaded error-suspect debug cycles are compared with the golden debug cycles. Thus, we can identify the exact erroneous debug cycles (13).

### Hierarchical MISR operation

**Fig 3:**
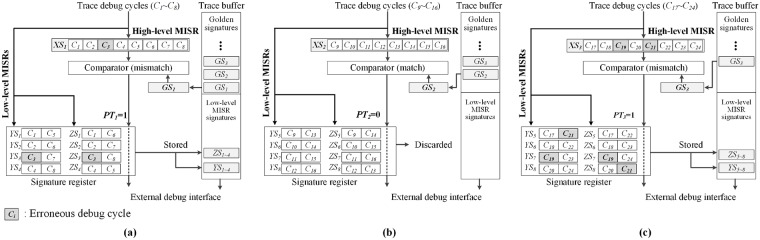
Hierarchical MISR operation: (a) and (c) cases of *PT*_*i*_ = 1; (b) case of *PT*_*i*_ = 0.

The proposed scheme comprises three MISRs as described in the previous section. The high-level MISR is used to directly compact the overall debug cycles. In this case, the consecutive debug cycles are compacted by the high-level MISR with the compaction ratio computed as the number of overall debug cycles divided by the trace buffer depth. In the proposed debug scheme, three types of signatures are generated by a hierarchical MISR. The concept of hierarchical MISR structure can be similar with the MISR composition in [11]. In the debug technique in [11], the two MISRs sample the consecutive debug cycles in different compaction ratios. And the segmented MISR signatures are only stored in the trace buffer. In our proposed debug scheme, the high-level MISR signatures are compared with the golden signatures that are prestored in the trace buffer by performing on-chip comparison. At the same time, the low-level MISRs generate the signatures newly by sampling the debug cycles which compose the error-suspect signature detected by on-chip comparison. We analyze the error-suspect debug cycles using only the low-level MISR signatures. This reduced the number of debug cycles that is required per signature for analyzing the error-suspect debug cycles. Fig 3 represents the overall operation of the hierarchical MISR. The high-level MISR generates the signature by compacting the consecutive debug cycles (*C*_*i*_). In this case, the number of samples per signature (SPS) is set to eight. The signatures generated by the high-level MISR (*XS*_*k*_) are transmitted to the on-chip comparator and are compared with the golden signatures (*GS*_*k*_) that are prepared in the trace buffer. In this case, *k* means the k-th generated signature. Simultaneously, the trace debug cycles are compacted by the low-level MISRs and are temporarily stored in the signature register. Further, the comparator generates a parent tag bit (*PT*_*k*_) as a result of the comparison. If *PT*_*k*_ is 1, the low-level MISR signatures that are stored in the signature register are transferred to the trace buffer. However, if *PT*_*k*_ is 0, the low-level MISR signatures are discarded, and the signature register is prepared to overwrite the stored low-level MISR signatures. The generated parent tag bit is also transferred to the external debugger. In this example, *C*_*3*_, *C*_*19*_, and *C*_*21*_ are assumed to be erroneous debug cycles. After completing the on-chip comparison of *XS*_*k*_ and *GS*_*k*_, the signatures *XS*_*1*_ and *XS*_*3*_ are observed to be different from the golden signatures, as depicted in Fig 3(a) and 3(c). According to the outputs of the comparator, *PT*_*1*_ and *PT*_*3*_ are denoted by 1. In this case, the trace debug cycles, *C*_*1*_-*C*_*8*_, and *C*_*17*_-*C*_*24*,_ compacted by low-level MISRs are stored in the trace buffer. Additionally, the area of *GS*_*k*_ that is already compared with the high-level MISR signature in the trace buffer can be overwritten to ensure the storage of low-level MISR signatures.

**Fig 4:**
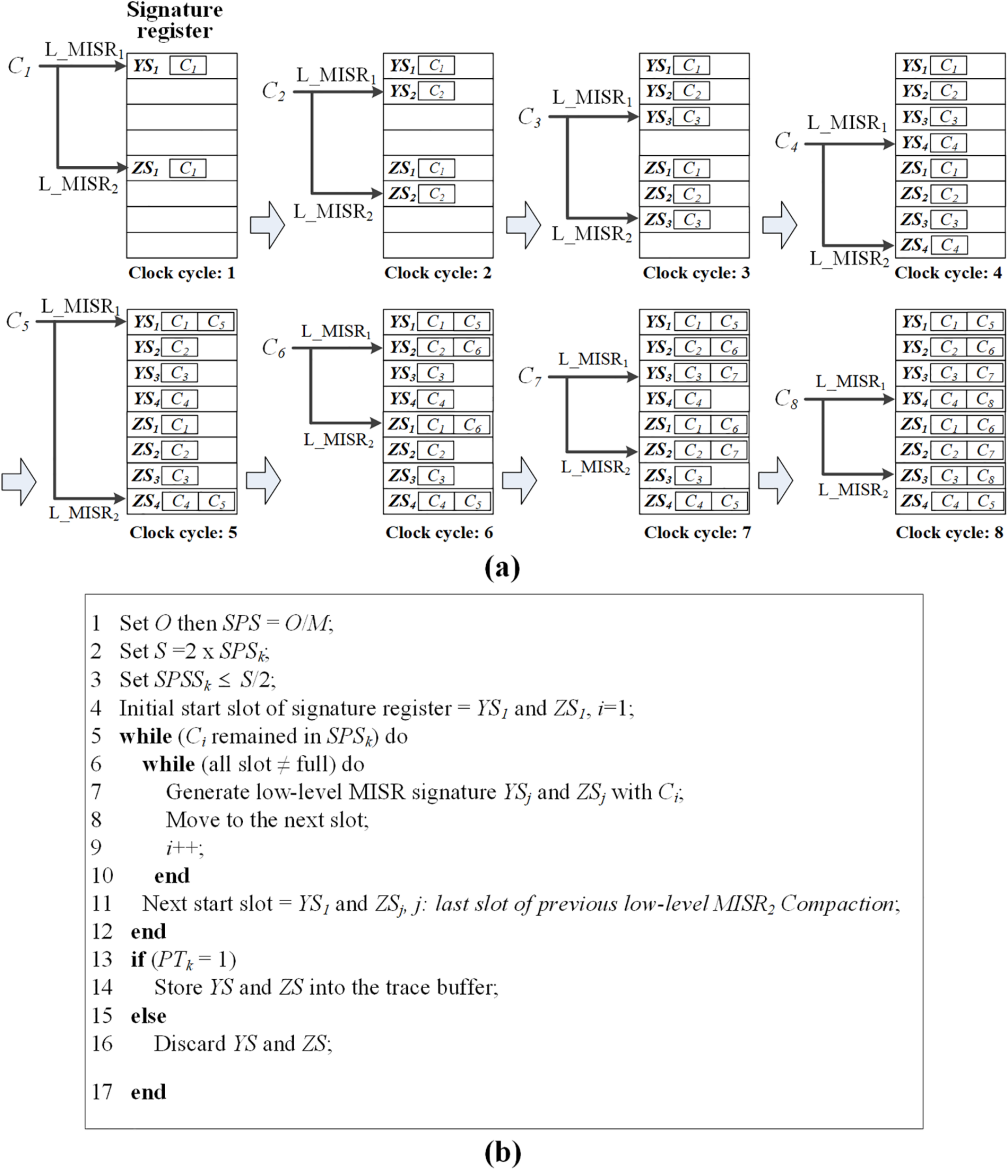
Low-level MISR operation to analyze error-suspect debug cycles: (a) temporary signature register usage for compaction of low-level MISRs; (b) algorithm for the operation of low-level MISRs and the signature register.

The low-level MISR signatures are actually stored in the trace buffer. Further, the low-level MISR signatures are unloaded via the external debug interface and are analyzed to detect error-suspect debug cycles by considering the commonly contained debug cycles between the signatures of the low-level MISRs. Fig 4 depicts the signatures generated by the low-level MISRs (*YS*_*j*_ for L_MISR_1_ and *ZS*_*j*_ for L_MISR_2_) and the analysis of the error-suspect debug cycles. The *ZS*_*j*_ of L_MISR_2_ is the delayed signature that is compacted at regular intervals from *YS*_*j*_. The trace debug cycles are compacted, and the generated signatures are stored in the signature register, as depicted in Fig 4(a). Additionally, the consecutively generated signatures are stored in different slots of the signature register. Thus, the maximum numbers of *YS*_*j*_ and *ZS*_*j*_ are ($M-\lceil \frac{N_{GS}}{W}\rceil )/2$. In this expression, *N*_*GS*_ is the number of golden signatures. *M* and *W* are the depth and width of the trace buffer, respectively. The algorithm to operate the signature register is presented in Fig 4(b). In the algorithm, *O* is the observation window, *M* is the trace buffer depth, *S* is the size of signature register, SPS is the number of samples per high-level MISR signature, and SPSS is the number of samples per low-level MISR signature, respectively. In this operation, we generate *ZS*_*j*_ using L_MISR_2_ as a complicated compaction. When the slots of the signature register are completely filled with the generated signatures, the slots are repeatedly filled with a new starting point. In this case, the starting slot points of *ZS*_*j*_ are different from those of *YS*_*j*_.

**Fig 5:**
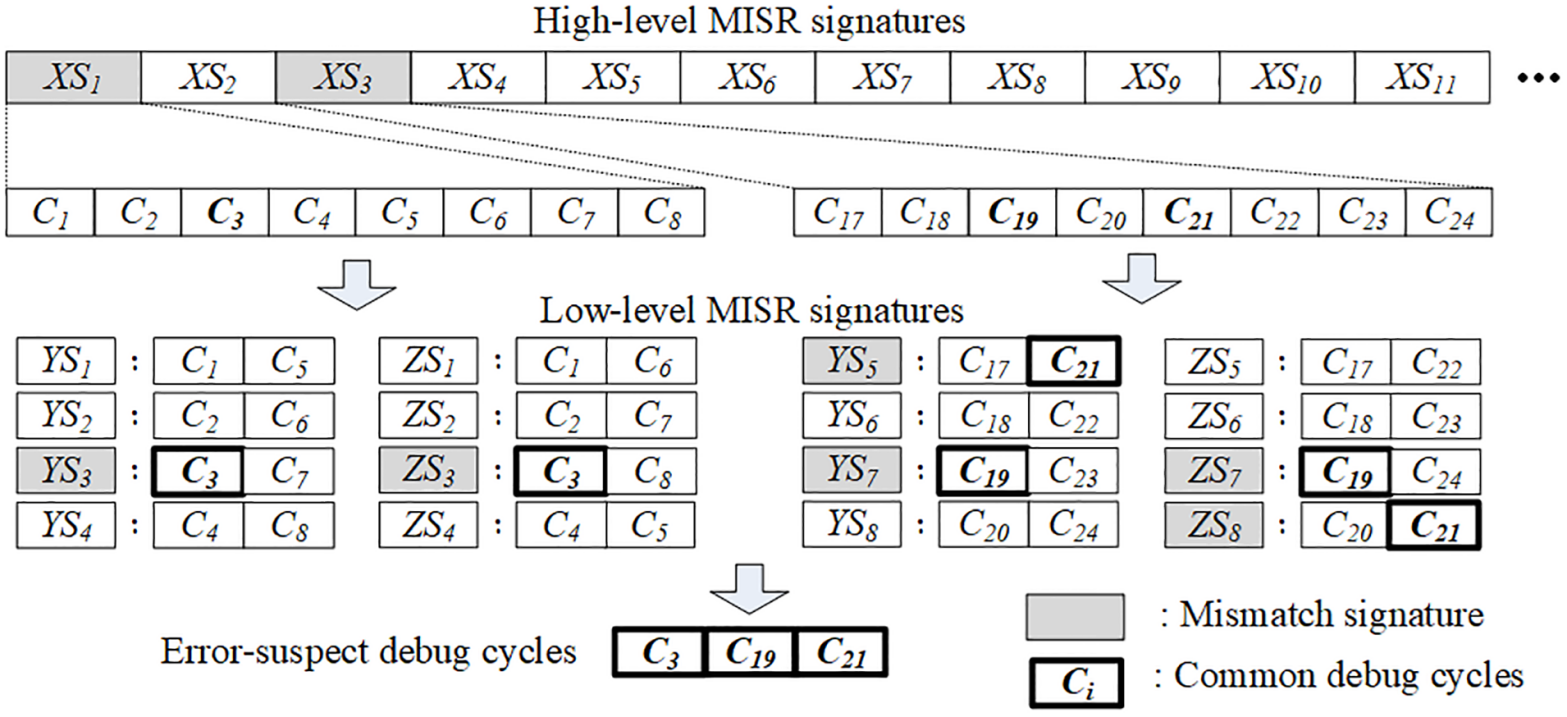
Error-suspect debug cycles after analyzing the common debug cycles in both *YS*_*i*_ and *ZS*_*i*_ that are generated by the low-level MISRs.

After the stored low-level MISR signatures are unloaded to the external debugger, the unloaded signatures are analyzed to identify the error-suspect debug cycles. Fig 5 depicts an analysis of the error-suspect debug cycles based on the flow of the debug process from the high-level MISR operation to the low-level MISR operation. A comparison of *XS*_*k*_ and *GS*_*k*_ depicts that there are two unmatched high-level MISR signatures, *XS*_*1*_ and *XS*_*3*_. After the low-level MISR signatures are unloaded, the unloaded *YS*_*i*_ and *ZS*_*i*_ are compared with the golden *YS*_*i*_ and *ZS*_*i*_ generated off-chip, and the erroneous signatures are classified. The erroneous *YS*_*i*_ and *ZS*_*i*_ are analyzed to determine the common debug cycles that are contained in both the erroneous *YS*_*i*_ and *ZS*_*i*_. In this example, the erroneous signatures are *YS*_*3*_, *YS*_*5*_, *YS*_*7*_, *ZS*_*3*_, *ZS*_*7*_, and *ZS*_*8*_, and the final error-suspect debug cycles that are identified to be *C*_*3*_, *C*_*19*_, and *C*_*21*_ are the common denominators of erroneous signatures.

### Capturing error-suspect debug cycles

**Fig 6:**
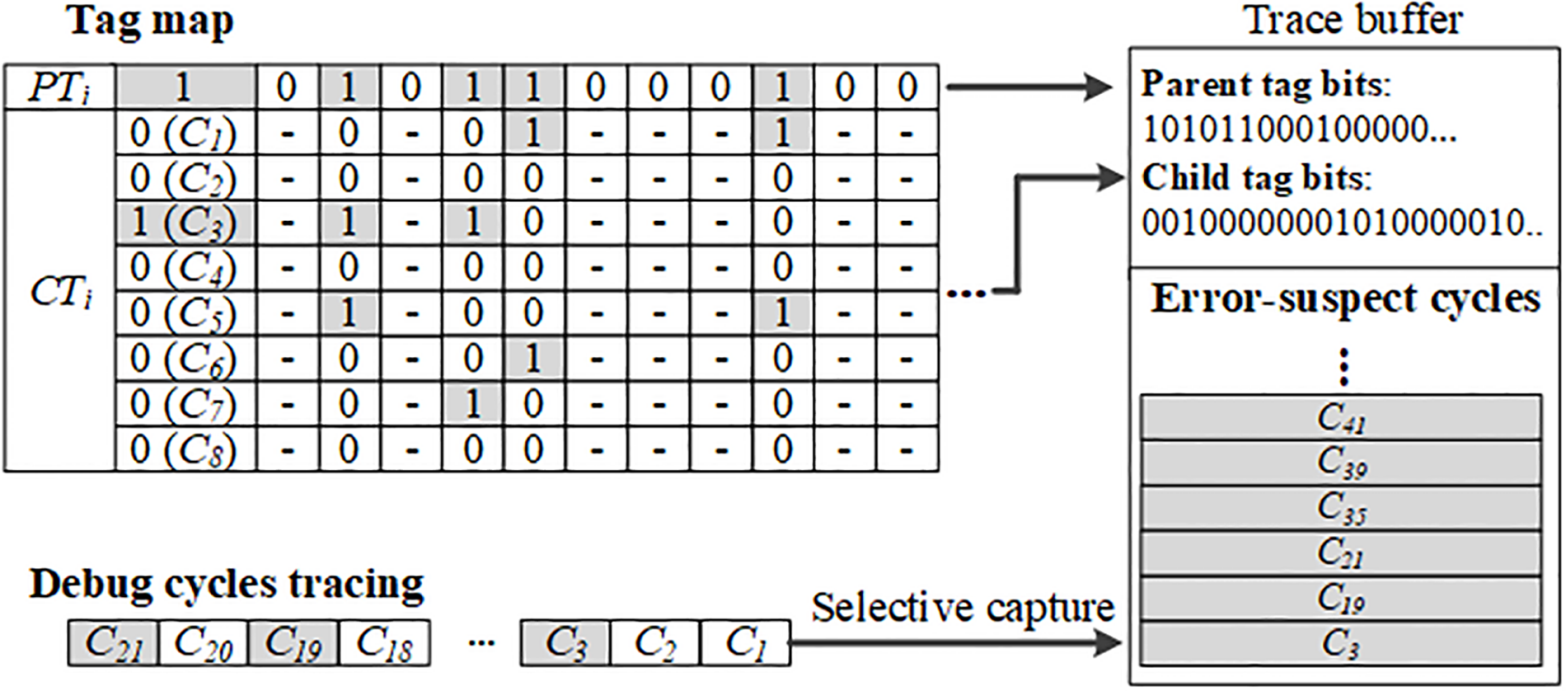
Usage of tag map and capturing of error-suspect debug cycles.

After completing the off-chip analysis of error-suspect debug cycles that use the low-level MISR signatures, the tag maps adopted from [13] are generated to selectively capture the error-suspect debug cycles. If the parent tag bit is one, the child tag bits corresponding to the error-suspect debug cycles are set to one. However, if the parent tag bit is zero, the child tag bits are not generated. Using the tag map, we determined the error-suspect debug cycles that should be captured. In the tag map depicted in Fig 6, the parent tag bits are generated as 101011000100, and the length of the child tag bits is observed to be eight. In addition, the child tag bits of *XS*_*1*_ are 00100000, which indicated that *C*_*3*_ is the error-suspect debug cycle. Similarly, the error-suspect debug cycles in the tag map are *C*_*19*_, *C*_*21*_, *C*_*35*_, *C*_*39*_, *C*_*41*_, *C*_*46*_, *C*_*73*_, and *C*_*77*_. The tag map is uploaded to the trace buffer after analyzing the error-suspect debug cycles. The error-suspect debug cycles are captured in the trace buffer when both the parent and the child tag bits are one. In this case, the sum of the allocated sizes of the captured error-suspect debug cycles and the tag map in the trace buffer should not exceed the size of the trace buffer.

### Hardware structure

**Fig 7:**
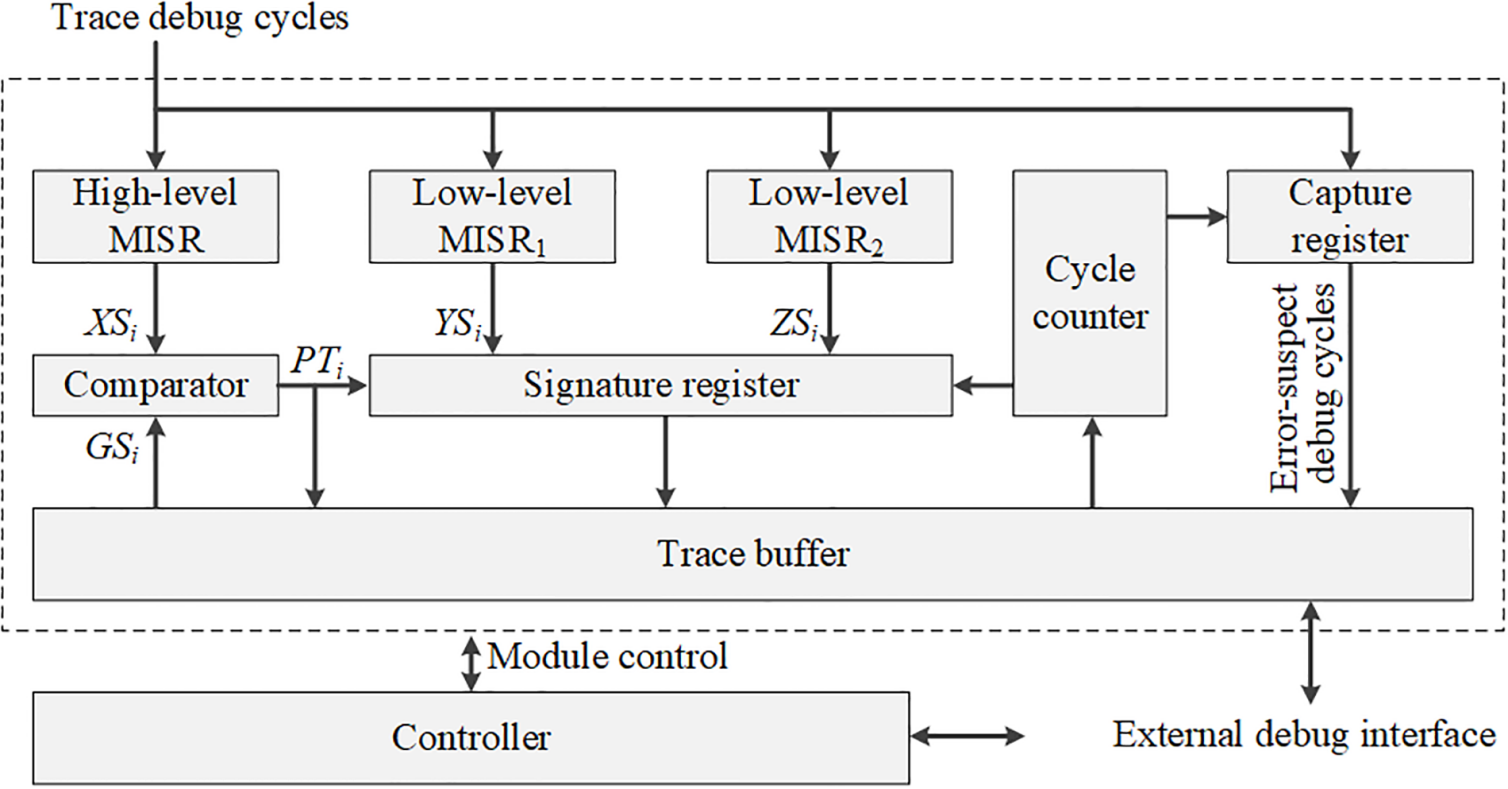
Hardware structure of the proposed debug scheme.

The proposed debug structure is represented in Fig 7. The debug control module is controlled through an external serial interface such as the TAP. The controller module controls the entire debug process, including the debug triggering and the trace buffer. In order to implement the MISRs in the proposed debug scheme, we can adopt the conventional MISR structure whose length is the same with the core output width. The low-level MISRs are controlled by the finite-state machine (FSM) to generate compacted signatures in desired sampling cycles. The low-level MISR signatures are temporarily stored in the signature register by controlling the FSM in the word counter. The number of signatures is stored in the cycle counter register. Therefore, the slot at which the trace debug cycles are compacted by the low-level MISRs and are stored based on the debug cycle number count. Additionally, the cycle counter triggers the capture points of the error-suspect debug cycles in accordance with the tag map information. The error-suspect debug cycles are captured in the capture register if both the parent and the child tag bits are one, and the error-suspect debug cycles are transferred to the trace buffer. The external interface is operated at a low frequency because of the TAP specification. Therefore, the low-level MISR signatures, the tag map, and the error-suspect debug cycles are transferred via the external debug interface by applying an operational frequency that is lower than the on-chip frequency.

## Results

In this section, we evaluate the proposed debug scheme and compare the proposed scheme with a previous method that is described in [12] based on the simulation results. The proposed debug structure is designed using the RTL code based on the OpenSPARC T2 core [14]. The OpenSPARC T2 core exhibits a 32-bit-wide data output. The duration of the overall tracing of the debug cycles (*N*) is 2^19^ cycles. Various trace buffer depths (128, 256, and 512) are used to perform simulations with five error rates (0.048%, 0.097%, 0.146%, 0.291%, and 0.488%). The error rates are estimated using *E*/*N*, where *E* is the number of erroneous cycles [12]. The system malfunction errors are injected in accordance with the uniformly random distribution within the overall tracing cycles. The area of the proposed structure is designed using a 130-nm application-specific integrated circuit (ASIC) standard cell library. Each experimental result is obtained by repeatedly simulating data for 1,000 times using the given debug conditions.

First, the error identification capability depends on the number of error-suspect debug cycles that is required to identify the exact erroneous debug cycles during the debug operation for the overall debug cycles. Table 1 depicts the normalized error identification capability in the 32-bit-wide core, which can be achieved using the proposed debug scheme. In this case, a lower value indicates better performance than other debug cases because fewer error-suspect debug cycles are required to identify the error data words. The proposed debug approach and the debug techniques in [12, 13] use the error-suspect debug cycles by analyzing the commonly contained debug cycles between the error-suspect signatures to identify the real erroneous cycles. On the contrary, the debug techniques in [9, 11] use the repeatable debug sessions to identify the real erroneous cycles. Therefore, the error-free cycles can be remained in the error-suspect debug cycles in the debug techniques in [12, 13]. By using the proposed debug scheme, the number of error-suspect cycles can be reduced to identify the erroneous cycles. For this reason, in Table 1, the required number of error-suspect cycles in the proposed debug scheme is compared with that in the debug techniques in [12, 13]. Generally, the proposed debug scheme requires fewer error-suspect debug cycles to identify the erroneous debug cycles than that achieved by the previous debug techniques. Our results, therefore, depict that the number of unnecessary error-suspect debug cycles can be reduced using hierarchical MISRs. Therefore, in our proposed method, the number of common debug cycles that are contained in the erroneous signatures generated by the high-level and low-level MISRs is observed to be less than that in the previous debug techniques.

**Table 1:** The required average number of error-suspect debug cycles for error identification (normalized).

Trace buffer depth	Error rates (%)	[12]	[13]	Proposed scheme
128	0.048	4.46	3.46	1.00
	0.097	6.26	7.06	1.67
	0.146	5.73	10.53	2.29
	0.291	10.54	21.37	5.07
	0.488	13.40	35.97	5.24
256	0.048	2.28	2.83	0.50
	0.097	3.28	5.78	0.84
	0.146	4.03	8.72	1.14
	0.291	5.47	17.57	2.56
	0.488	7.06	29.68	2.63
512	0.048	1.27	2.42	0.25
	0.097	1.71	5.13	0.26
	0.146	2.09	7.74	0.58
	0.291	2.87	15.61	1.29
	0.488	3.62	26.51	1.34

To evaluate the overall debug time, the total amount of debug data is categorized based on internal tracing and external transmission during the overall debug process. Further, the overall debug time is given by *N*_*in*_ × 1/*f*_*in*_ + *N*_*ex*_ × 1/*f*_*ex*_, where *f*_*in*_ denotes the internal operational frequency, *N*_*in*_ denotes the total number of trace debug cycles, and *N*_*ex*_ denotes the number of debug data cycles that are transferred between the trace buffer and the external debugger during the overall debug process. The external frequency is given by *f*_*ex*_ if TAP is used to transfer the debug data between the trace buffer and the external debugger. In this case, the ratio between the internal and external operation frequencies is assumed to be 10:1. It is usually difficult to temporarily pause the data-tracing process during the hardware debug. Therefore, data tracing should be reset to the initial state after completing one debug process within the observation window. Therefore, we repeat the front observations of the debug order until the debug operations of all of the trace data are terminated.

Table 2 presents the debug time reduction ratios when the compaction ratio of the high-level MISR is fixed in all of the debug cases. A reference debug time is acquired when the conventional observation window is applied without any MISR compaction. The proposed debug scheme requires less debug data than that required in [12] in the equal observation window. Therefore, the debug time of the proposed method is also less than that observed in [12], and the debug time reduction ratio of the proposed method is higher than that observed in [12]. If the error rate is increased, more debug data are required to identify the erroneous debug cycles. This reduces the reduction ratio. However, if the trace buffer depth is increased, the number of repeatable debug segments and the debug time is also reduced.

**Table 2:** Debug time reduction ratio in the same observation window.

Trace buffer depth	Error rates (%)	Reduction ratio (T_conv_/T_prop(prev)_)				
		[9]	[11]	[12]	[13]	Proposed scheme
**128**	0.048	39.71	59.94	8.30	8.44	43.44
	0.097	32.77	46.18	7.09	6.33	33.94
	0.146	23.36	37.41	6.62	5.01	28.25
	0.291	14.92	24.91	5.73	2.97	19.63
	0.488	11.90	18.00	4.57	1.95	15.05
**256**	0.048	26.48	32.87	8.49	9.34	45.46
	0.097	20.88	33.44	8.26	7.76	40.26
	0.146	15.69	25.21	8.04	6.57	34.23
	0.291	9.25	11.65	7.04	4.45	25.43
	0.488	6.64	6.15	6.59	3.09	21.49
**512**	0.048	24.98	25.88	9.45	10.03	76.49
	0.097	20.16	25.83	8.90	8.54	36.05
	0.146	14.38	25.83	8.37	7.84	33.97
	0.291	9.40	17.35	7.96	5.74	27.27
	0.488	6.45	13.11	7.16	4.31	23.08

Table 2 also shows the experimental result for the comparison of the debug time reduction ratio of the proposed debug scheme to the debug techniques in [9, 11, 13]. While the small trace buffer depth is used to get the result of the debug experiment, the debug time reduction ratio of the debug technique in [11] is larger than that of other debug techniques. In this case, the SPSS of the proposed debug approach is relatively higher than that of [11] as the debug operation is progressed. For this reason, the debug time reduction ratio of the proposed debug scheme is less than that of [11] in small buffer size. On the contrary, the debug time reduction ratio of the proposed debug scheme is larger than that of other debug techniques as the trace buffer size increases. In this case, the trace buffer size in the proposed debug scheme is sufficient to generate the low-level MISR signatures. For example, the number of error-suspect debug cycles in the proposed debug scheme is less than that in the debug techniques in [9, 11] when 512-depth buffer is applied to the debug operation. For this reason, the debug time reduction ratio is reduced in the large trace buffer usage due to longer transferring time of debug data via the external debug interface.

Table 3 presents the hardware area overheads in terms of the two-input NAND equivalent gates. This table depicts a comparison between the previous debug technique and our proposed debug structure applied to the OpenSPARC T2 core. In this case, the area overheads of the debug modules are only included in the results because the trace buffer size applied in the proposed debug approach is the same as that of previous works. The hardware area overhead of the proposed debug structure is 1.91% of that of the OpenSPARC T2 core. The hardware area overheads for the debug techniques in [9, 11, 13] also have been evaluated. As shown in Table 3, the hardware area overhead of the proposed debug scheme is larger than that of other debug techniques. In this case, the number of MISR and the size of the signature register affect the hardware area overhead of the proposed debug scheme. However, the hardware area overhead of the proposed debug scheme is still much less than the processing core.

**Table 3:** Hardware area overhead.

Design	OpenSPARC T2	Debug module	Overhead (%)
**[9]**	595,738	2,037	0.34
**[11]**		3,450	0.58
**[12]**		9,124	1.53
**[13]**		10,783	1.81
**Proposed**		11,395	1.91

## Conclusions

In this study, we propose a debug scheme that can reduce the amount of debug data that is required to identify the erroneous debug cycles. We can obtain a better error identification capability using hierarchical MISRs for decreasing the commonly contained error-suspect debug data from among the generated MISR signatures. Therefore, we can decrease the unnecessary debug data transfer from debug operations. All these factors reduces the debug time, and the proposed debug scheme accelerates the post-silicon validation.
